# Effects of protein energy supplementation during pregnancy on fetal growth: a review of the literature focusing on contextual factors

**DOI:** 10.3402/fnr.v57i0.20499

**Published:** 2013-11-12

**Authors:** Selma C. Liberato, Gurmeet Singh, Kim Mulholland

**Affiliations:** 1 Menzies School of Health Research, Charles Darwin University, Darwin, Australia; 2 Northern Territory Medical Program, Flinders University, Adelaide, Australia; 3 Departments of Epidemiology and Public Health, London School of Hygiene and Tropical Medicine, London, UK

**Keywords:** maternal supplements, protein energy supplements, fetal growth, intrauterine growth, infants, birth weight

## Abstract

**Background:**

Maternal diet during pregnancy is one of the most important factors associated with adequate fetal growth. There are many complications associated with fetal growth restriction that lead to lifelong effects. The aim of this review was to describe the studies examining the effects of protein energy supplementation during pregnancy on fetal growth focusing on the contextual differences.

**Methods:**

Relevant articles published between 2007 and 2012 were identified through systematic electronic searches of the PubMed, Science Direct, and EBSCO database and the examination of the bibliographies of retrieved articles. The search aimed to identify studies examining pregnant women receiving protein and/or energy during pregnancy and to assess fetal growth measures. Data of effectiveness and practical aspects of protein energy supplementation during pregnancy were extracted and compiled.

**Results:**

Twenty studies (11 randomized controlled trials, 8 controlled before and after, and 1 prospective study) were included in this review. Positive outcomes in infants and women cannot be expected if the supplementation is not needed. Therefore, it is essential to correctly select women who will benefit from dietary intervention programs during pregnancy. However, there is currently no consensus on the most effective method of identifying these women. The content of protein in the supplements considering total diet is also an important determinant of fetal growth. Balanced protein energy supplementation (containing up to 20% of energy as protein) given to pregnant women with energy or protein deficit appears to improve fetal growth, increase birth weight (by 95–324 g) and height (by 4.6–6.1 mm), and decrease the percentage of low birth weight (by 6%). Supplements with excess protein (>20% of energy as protein) provided to women with a diet already containing adequate protein may conversely impair fetal growth. There is also no consensus on the best time to start supplementation.

**Conclusions:**

Strong quality studies examining adequate criteria to screen women who would benefit from supplementation, time to start supplementation, and type of supplements are warranted.

Low birth weight (LBW) is a major problem throughout the developing world. In the Middle East/North Africa, 15% of infants are born with low weight (1, 2). In sub-Saharan Africa, the proportion is 14% (1), ranging from 13.5% in east Africa to 17% in West Africa (3). South Asia has the highest incidence, with a rate of 31% of all infants, whereas East Asia/Pacific has the lowest, at 7%. India is home to nearly 40% of all LBW babies in the developing world (2).

Maternal diet during pregnancy is one of the most important factors associated with infants’ birth weight and thus birth weight has often been used as an indicator of woman's nutrition during pregnancy. Better health outcomes for both infants and their mothers are seen when infants are born at term (between 37 and 42 weeks of gestation) and weighing between 2,500 and 4,000 g. On the contrary, both prematurity (born before 37 weeks of gestation) and LBW (<2,500 g) are associated with significant complications, including respiratory distress syndrome, pneumonia, infection, apnea, bradycardia, anemia, and jaundice. The earlier the gestational age and the lower the birth weight, the greater the risk of complications (4).

Birth weight is commonly available and makes LBW a convenient measure to use as an indicator of maternal health. However, to adequately discriminate between preterm and growth-retarded babies, gestational age is also required. While a cut-off of 2,500 g is adequate to differentiate the growth of most term babies, all preterm babies, whether they are normally grown or growth restricted, will be classified as LBW. A better indicator of fetal growth is small for gestational age, which is defined as birth weight < 10th percentile for gestational age. The outcomes for the infants are worse when fetal growth restriction (FGR) rather than prematurity is the cause of LBW. There are many complications associated with FGR that lead to lifelong effects, including the risk of renal disease, cardiovascular disease, and diabetes (5) and infants born with FGR are 5–10 times more likely to die in the first year of life than are average gestational age infants (6). It is estimated that 11% of infants in low-income countries are born with FGR (6).

Some studies have shown that supplements of more than 2,920 kJ (700 kcal)/day (7) and containing up to 25% of energy as protein (8) provided to women during pregnancy reduce the risk of a LBW baby by 32% in certain contexts (7). On the contrary, there are studies showing no or even deleterious effects of protein energy supplements (9, 10). The aim of this review was to describe these studies to better understand these contradictory findings on effect of protein energy supplementation on birth outcomes including any anthropometric measurements (birth weight and height, head circumference) and prevalence of LBW or small for gestational age considering the contextual differences.

## Methods

### Literature search

**Fig. 1:**
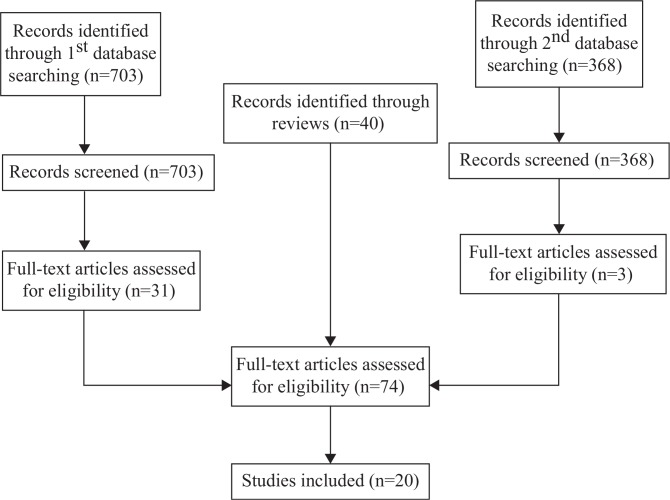
Flow diagram.

The PubMed, EBSCO, and Science Direct database were searched on May 14, 2010, using the following search terms ‘food supplement*’, ‘energy supplement*’, ‘protein supplement*’, and ‘pregnan*’ in the abstract field, and 703 studies published between 2007 and 2010 were retrieved. Screening based on title and abstract as judged by one author (SCL) reduced the number to 31 articles. Four reviews (7, 8, 11, 12) were also retrieved and all relevant studies from the reference lists were identified. The reviews included studies published up to 2007 and, therefore, the search of the database was restricted to studies published after 2007. A total of 71 studies were identified as relevant and 69 full papers were obtained and read. The database was searched again on Jan 26, 2012, using the same key words and 368 references between 2010 and 2012 were retrieved. Screening based on title and abstract as judged by the same author (SCL) reduced the number to three articles. After reading the full paper, two articles were included (Figure 1).

### Inclusion/exclusion criteria

The following inclusion criteria were used to identify studies: 1) the subjects were pregnant women, 2) protein and/or energy were the only components of the supplement that differed between treatment groups, and 3) fetal growth measures such as birth weight and head circumferences were reported. When more than one publication about the study was found, only one publication reporting more contextual factors and/or outcomes of interest was included. A total of 20 studies met the inclusion criteria.

There was no restriction on the criteria used to screen women who would benefit from supplementation or type of food provided.

Reasons for excluding studies included:

- Inclusion criteria to select participants is not reported or different inclusion/exclusion criteria for control and intervention group
- No control group
- Problems in the availability of supplementation
- Effect of supplementation during postpartum rather than during pregnancy was investigated
- Supplementation was not the only component of the intervention e.g. nutrition education is included
- Measurements of birth weight not reported

### Data abstraction

Data extracted from each eligible study included the following variables: study context, criteria to screen participants, intervention specifics, and outcome effects.

## Results

Twenty studies examining the effects of protein energy supplementation during pregnancy on fetal growth were included in this review. Eleven were randomized controlled trials, eight were controlled before and after, and one was a prospective study (Table 1).

**Table 1:** Setting, screening criteria, intervention, and main outcomes of studies examining supplementation during pregnancy on fetal growth

References	Place	Criteria used to screen participants into the study	Design and intervention	Main outcomes
Adams et al. (13)	San Francisco	At least one of the following: systolic 140 and/or diastolic 90 mmHg; fetal loss; heavy smoking; heart disease; not married to biologic father at conception; height < 157.5 cm; or birth weight (BW) is 15% or more below standard weight for height.	Randomized controlled trial (RCT), 102 women by week 27 of gestation received daily supplementation of: 40 g protein + 1,960 kJ (470 kcal), vitamin and minerals (34% of protein) 6 g protein + 1,338 kJ (320 kcal) + vitamin and minerals (7.5% of protein) Vitamin and minerals	No difference in BW of infants between the treatment groups.
Blackwell et al. (14)	Taiwan	Maternal protein intake < 40 g/d, Hg > 11 g/100 mL, hematocrit > 36% and plasma protein > 5.5 g/100 mL	Prospective study, 294 women in the third trimester of their second or third pregnancy receiving daily: Mineral and vitamin supplements 3,344 kJ (800 kcal), 20% of protein, mineral and vitamin.	Among those second study infants, there was no difference between infants’ BW born to women receiving only mineral and vitamin and those born to women receiving mineral and vitamin and 3,340 kJ (800 kcal). Male infants born to supplemented women were heavier (3,197 g) and longer (49.77 cm) than those born to unsupplemented women (3,062 g and 49.16 cm, *p*<0.05).
Brown (15)	Aberdeen, UK	Two or more of the following indices in the lowest quartile: weight at 20 weeks of gestation, height, weight gain and weight for height at 20 weeks	RCT stratified to village size, 1,056 pregnant women at 20 weeks gestation received daily: Control Flavored milk providing 1,220 kJ (293 kcal), 20.5% protein or fresh milk providing 1,620 k J (387 kcal), 20.7% protein or cheddar cheese providing 1,330 kJ (319 kcal), 23.8% protein	Supplementation resulted in higher maternal weight gain after 30 weeks of gestation No significant difference in infants’ BW but there was positive correlation between protein and energy and BW
			7 d weighted record and urinary nitrogen were collected.	
Ceesay et al. (16)	Gambia	None	RCT, 1,460 women at week 20 of pregnancy who gave birth to 2,047 singleton live births, plus 35 stillbirths during the study period non-selected but living where food shortage happens during wet season received daily supplementation: Control (supplement provided for 20 weeks after delivery) 4,250 kJ (1,017 kcal), 8.5% protein) provided from around 20 weeks of gestation.	Supplementation increased BW and head circumference throughout the year with greater increases in the hungry season than in the harvest season. The percentage of low birth weight (LBW) was lower in the intervention group (11.1%) than in the control group (17%) throughout the year (*p <*0.01).
			All women received iron and folate supplements according to their hemoglobin concentration and a weekly prophylactic dose of chloroquine during the hungry season.	
Huybregts et al. (17)	Burkina Faso	None	RCT, 1,296 non-selected pregnant women received daily: Multiple micronutrient supplement; Multiple micronutrient + spread with 1,560 kJ (373 kcal) and 15.8% of protein	Mean birth length of infants born to women supplemented with spread containing protein and energy in addition to multiple micronutrients was 4.6 mm (*p =*0.01) higher compared to that of infants born to women supplemented only with multiple micronutrients in multigravid women. No significant difference was observed for primigravid women. In women with body mass index (BMI) < 18.5 at enrolment, the difference in mean birth length of infants born to women supplemented with spread and multiple micronutrients was 12.0 mm (95% CI: 3.7, 20.2; *P =*0.005) greater compared to the length of infants born to women supplemented only with multiple micronutrients.
Iyenger (18)	India	Low socioeconomic status	Controlled before and after (CBA), 25 women at 36 weeks of gestation receiving daily: Diet at hospital with 8,780 kJ (2,100 kcal), 60 g protein (11.4% of protein) Diet at hospital with 8,780 kJ (2,100 kcal), 60 g protein added of 35 g of protein (40% of protein) Non-hospitalized women having normal diet of 5,850 kJ (1,400 kcal), 40 g of protein	Higher infants’ BW born to supplemented women (either added of 35 g or protein or not) (3,028±83 g) compared to those born to unsupplemented women (2,704±24 g) (*p <*0.01). No difference in the BW of infants born to women receiving supplementation and those born to women receiving supplementation added of 35 g of protein.
			All women received daily iron and multivitamin supplements.	
Kardjati et al. (19)	East Java	Women living in areas know to be nutritionally vulnerable	RCT, 741 women in week 26–28 of gestation received daily: Low protein [1,940 kJ (465 kcal), 10% of protein] High protein [220 kJ (52 kcal), 50% of protein]	No difference in BW between the groups. The authors mention that better home diet during the experimental period may have masked the effect of maternal supplementation on infants’ BW.
Khan et al. (20)	Bangladesh	Pregnant women < 14 weeks confirmed by ultrasound examination, no severe illness and with viable fetus.	RCT, 4,436 women received daily food supplementation (2,540 kJ (608 kcal), 11.8% of protein) either immediately after identification of pregnancy or later (usually in the second trimester) added of: 30 mg Fe and 400 µg folic acid (Fe30F) 60 mg Fe and 400 µg folic acid (Fe60F) Multi mineral and vitamin supplementation.	The proportion of LBW did not differ across the intervention groups. There was no significant difference in mean weight-for-age, weight-for-height, or height-for-age across intervention groups. Early invitation to prenatal food supplementation to pregnant mothers resulted in reduced proportion of stunting. Among mothers with higher BMI (BMI ≥ 19.7) stunting was less frequent (difference 4.6%, 95% CI = 0.1 to 9.1%, *p*=0.05) in mothers supplemented immediately after identification in comparison with late food supplementation, while this was not significant among mothers in the lowest half of the BMI distribution (difference 4.3%, 95% CI = − 0.6 to 9.2%).
			The anthropometry of 3,267 children was followed from birth to 54 months, and 2,735 children were available for analysis at 54 months.	
Mardones-Santander et al. (21)	Chile	Low socioeconomic status and underweight (<95% standard at week 12 of gestation)	RCT, 597 women before 20 weeks of pregnancy who had full term without complications received daily: Powdered milk containing 2,080 kJ (498 kcal), 27.9 g of protein, (22.4% of protein) Milk-based fortified product containing 1,960 kJ (470 kcal), 14.5 g protein (12.3% protein).	Higher BW (3283.3 g) in infants born to women supplemented with milk based fortified product containing 12.3% of protein compared to BW (3219.8 g, *p <*0.05) of infants born to women supplemented with powdered milk containing 22.4% of protein. Lower percentage (32%) of small for gestational age infants among infants born to women supplemented with milk based fortified product containing 12.3% of protein compared the percentage (44%) of small for gestational age infants born to women supplemented with powdered milk containing 22.4% of protein (*p <*0.05).
McDonald et al. (22)	Taiwan	Lowest rank of socio economic status and ‘nutritionally at risk’ due to low protein and energy intake [with daily intake of 5,020 kJ (1,200 kcal) and < 40 g of protein]	RCT, 213 multigravid women who had two children during the 6.5-year study period received daily supplementation from 3 weeks after the birth of a first study infant and continued throughout lactation, and through to the end of lactation of a second study infant: 3,340 kJ (800 kcal, 20% protein were protein) plus vitamin and mineral supplements Control [<330 kJ (80 kcal), no protein plus vitamin and mineral supplements].	Second male infant born when woman had supplementation had higher BW (161.4 g, *p <*0.05) than first male infant born when the women did not have supplementation. This difference was not significant among female infants or control group
Mora et al. (23)	Colombia	Living in poor Southern barrios of the city	RCT, 456 women at week 28 of gestation receiving daily: Control (no supplement) 3,580 k J (856 kcal), 18% of protein, iron and vitamin A supplements	Supplementation increased male infants’ BW Higher BW (by 95 g, *p <*0.05) in full term male infants born to supplemented women compared to control group. Higher BW (by 105 g, *p <*0.05) in full term male infants born to women supplemented for 13 weeks or more to compared to control group.
Nahar et al. (24)	Bangladesh	None, but women with BMI < 18.5 received supplementation since first presentation while women with BMI > 18.5 started at 4 months until the end of pregnancy	CBA, 1,104 non-selected women at 2nd up to 6th month of pregnancy Daily supplementation: 3,340 kJ (800 kcal), 12% protein	There was no difference in mean BW of infants born to mother with BMI < 18.5 supplemented or not. Compared to women with BMI > 18.5, those with BMI < 18.5 had higher rates of LBW infants, irrespective of supplementation status.
Osofsky (25)	Philadelphia	Low socio economic status residing in an urban poverty area	CBA, 240 women at week 28 of gestation received Twice daily protein mineral supplementation (1,050 kJ; 250 kcal), 20 g protein, vitamin A, vitamin C, vitamin K, Ca, Mg, P and Na (32% of protein) Normal diet (control group) Diet intake was assessed by 24-h dietary recall at 2 weeks intervals up to four times	Nutritional analysis showed that the group was not nutritionally deprived and protein accounted to 14.8% of the energy intake. Lower BW (2,968 g) and height (49.0 cm) of infants born to women receiving protein mineral supplementation compared to those born to women in the control group (3,080 g and 50.0 cm, *p <*0.05)
Prentice et al. (9)	Gambia	None	CBA using retrospective controls, 197 singleton infants born during 4 years of supplementation intervention, and 182 singleton infants born in the 4 years immediately before the intervention (control) whose mothers received daily supplementation 3,970 to 4,600 kJ (950 to 1,100 kcal) at discretion of participants, having 14.5% of protein.	When the women were in negative energy balance, supplementation increased mean BW. When the women were in positive energy balance, the supplementation had no effect on birth outcomes.
Rasmussen & Habicht (26)	Panama	None	CBA, 520 non-selected women at third trimester of gestation of the first pregnancy up to 8 years received daily: Atole [3,800 kJ (910 kcal)/L containing 28% of protein and micronutrients] Fresco [1,380 kJ (330 kcal)/L containing 100% of carbohydrate and micronutrients]	Fresco was 3 times more consumed than Atole resulting in similar energy intake with the 2 supplements. Higher intake of vitamin and minerals among those women consuming Fresco. Higher consumption of either supplements was associated with an overall increase in infants’ BW and decrease in the% of LBW infants from 18% to 9% Those women with lower fat stores (lower skinfold thickness) and those who consumed higher amounts of supplements continuously from one pregnancy to the next had infants with higher BW.
Ross et al. (27)	South Africa	Black women	RCT, 127 women at 20 weeks of pregnancy received daily: Placebo; Zinc (30 to 90 mg) High bulk supplement: mixture of beans and maize + vitamins accounting for 3,240 kJ (776 kcal), 36 g protein (18.6% protein) Low bulk supplement: skim milk, maize flour, vitamin and minerals accounting for 2,930 kJ (700 kcal) and 44 g of protein (25% protein).	Higher BW (3,376 g) in infants born to mothers receiving low bulk supplementation compared to those born to unsupplemented mothers (3,177 g, *p <*0.05). BW (3,376 g) of infants born to mothers receiving low bulk supplementation was also higher than BW of infants born to women supplemented with zinc (3,088 g, *p*< 0.001) or with high bulk supplementation (3.082 g, *p <*0.005).
Rush et al. (28)	New York	Indigent, black women with < 63.7 kg, having at least one of the following: pre-pregnant weight < 50 kg, low weight gain, <50 g of protein intake in the last 24 h, and at least one previous LBW infant.	RCT, 770 women before week 30 of pregnancy receiving daily: Control (only vitamin and mineral supplements) High protein [40 g ptn, 1,960 kJ (470 kcal), 34% protein + vitamin and mineral supplements] Low protein [6 g ptn, 1,350 kJ (322 kcal), 7.5% protein + vitamin and mineral supplements].	No difference in the BW of term infants among the groups. Among infants born prematurely, high protein supplements produced growth restriction. The BW of infants born to women supplemented with high protein was lower (2,254 g) than that of infants born to women supplemented with low protein (2,577 g, *p <*0.02) or with only vitamin and minerals (2,587 g, *p <*0.01) The 13 women in the high protein group who delivered before 33 week of gestation consumed more supplement and fewer total calories (*p <*0.05) compared to women in the same group who delivered later.
Viegas et al. (29)	Birmingham, Asia	Increase in the triceps skinfold during second trimester < 20 µm/week	CBA, 45 mother by week 20 of gestation received daily supplementation of: Vitamins Vitamins + carbohydrate [42–125 MJ (10,000 to 30,000 kcal)/trimester] Vitamins + carbohydrate [42–125 MJ (10,000 to 30,000 kcal)/trimester) + protein (5 to 10% of energy intake]	Infants born to women supplemented with vitamin + carbohydrate + protein were 310 g heavier (*p <*0.05) compared to infants born to women supplemented with only vitamin.
Viegas et al. (30)	Birmingham, Asia	None	CBA, 153 mother by week 20 of gestation received daily supplementation: Vitamins Vitamins + carbohydrate [42 to 80 MJ (10,000 to 19,000 kcal/trimester)] Vitamins + carbohydrate [42 to 80 MJ (10,000 to 19,000 kcal)/trimester) + protein (5 to 10% of energy intake)]	No difference in BW of infants between the treatments.
Villar & Rivera (31)	Panama	Non-selected women from a place with high level of malnutrition	CBA, 169 pregnant women in the 2nd or 3rd trimester from the first child receiving daily: Atole [681 kJ (163 kcal), 28% of protein] Fresco [209 kJ (50 kcal), 100% as carbohydrate].	Women in the HHH group, receiving the highest amount of supplements had first infants with higher BW (3,799 g± 515 g) compared to all the other groups (2,855 g±471 g; 3,073 g±429 g and 2,969 g±424 g for LHH, LLH and LLL, respectively, *p <*0.01). Women in the HHH group had the second infants with higher BW (3,290 g±514 g) compared to all the other groups (LHH = 3,150 g±474 g, LLH = 3,056 g±378 g and LLL = 2,944 g±501 g, *p <*0.025). The difference in BW (150 g, *p <*0.05) was also significant between LHH and LLL groups when adjusted for parity, maternal height, and BW of the first child.
			Four groups were formed according to the supplement intake during first pregnancy, lactating period and second pregnancy: HHH [women consumed > 83.6 MJ (20,000 kcal) during each pregnancy and > 167.2 MJ (40,000 kcal) during the interim period], HHL [women received > 83.6 MJ (20,000 kcal) during first pregnancy,>167.2 MJ (40,000 kcal) during the interim period and < 83.6 MJ (20,000 kcal) during the second pregnancy], HLL and LLL.	

A list of the 15 excluded studies and reasons for exclusion is presented in Table 2.

**Table 2:** Excluded references and reason for exclusion

References	Reason for exclusion
Atton & Watney (32)	Different criterion to select control and intervention group. Included criteria for intervention group included Asian or BMI < 20, <50 kg or previous history of small babies, or late miscarriages, or premature labor. Women were included in the control group if they presented none of the above characteristics.
Balfour (33)	No sufficient food for all participants due to financial problems
Caan et al. (34)	Intervention and control groups received postpartum supplementation for 5–7 and 0–2 mo, respectively. Both intervention and control group received same type of supplementation during pregnancy.
Dieckmann et al. (35)	Selection criteria to include participants is not mentioned
Ebbs et al. (36)	No statistical analysis
Elwood et al. (37)	There were many problems during the intervention such as delay with the tokens and the supplement was provided half of the duration of the pregnancy.
Kardjati et al. (38)	No additional information than that provided in Kardjati (19)
Kardjati et al. (39)	No additional information than that provided in Kardjati (19)
Kusin et al. (40)	No additional information than that provided in Kardjati (19)
Martorell et al. (41)	No additional information than that provided in Villar & Rivera (31)
Moss & Carver (42)	Outcome of interest is not reported
Prentice et al. (43)	No additional information than that provided in Prentice et al. (9)
Rush (44)	Intervention included nutrition education and supplementation during pregnancy
Schramm (45)	Duration of supplementation, vitamin and mineral supplementation, monitoring of food supplement consumption and birth weight are not reported.
Stockbauer (46)	Duration of supplementation, vitamin and mineral supplementation and monitoring of food supplement consumption are not reported
Tofail et al. (47)	There is no control group as all participants received supplements.

### Criteria to screen women for dietary intervention programs

It is difficult to compare studies due to different screening procedures. It would be unrealistic though to have a universal consensus, as it may not be possible to apply the same screening for women recruited in different contexts. Some studies (Table 1) have used no criteria (16, 17, 26, 30, 43) while other studies have used a range of criteria including:

- Socioeconomic status: low socioeconomic status (18, 21, 22, 25, 28), living in areas known to be nutritionally vulnerable (19, 23);
- Race: black people (27, 28);
- Body composition: maternal body weight (13, 21, 28), body mass index (BMI) (24), and increase in triceps skinfold (29)
- Maternal diet: energy balance (22), protein intake (14, 22, 28); and
- Medical history: at least one previous LBW infant (28) or fetal loss history (13).

Differences in criteria and cut-offs used to screen pregnant women could explain different outcomes of protein energy supplementation during pregnancy on infants and women reported by different studies investigating this effect. Positive outcomes in infants and women cannot be expected if the supplementation is not needed. On the contrary, there is a margin of energy deficiency below which fetal growth is affected (38) and positive women and infants’ outcomes are likely to occur when supplementation is provided to women whose diet is not providing enough energy and nutrients. There is no further information on level of energy deficiency that affect fetal growth.

Five studies (Table 1) examining the effects of protein energy supplementation on birth weights (of infants) during pregnancy have used no criteria to screen women who would benefit or not over-consume from supplementation. Increased birth weight of infants was observed when women in negative energy balance were supplemented during pregnancy (16, 17, 43), and the difference was greater during ‘the hungry season’, the time of the year when food is scarce (16), or among women who had a BMI < 18.5 (17) compared to control group. There was only a modest effect (48) or no effect (16) when food was readily available (harvest season). These findings show that the use of screening criteria may be unnecessary in areas with high levels of malnutrition, such as some African countries.

Socioeconomic status or living conditions may provide a good indication of women who would benefit from supplementation during pregnancy. Five (18, 21–23, 31) of eight studies using low socioeconomic status criteria found higher birth weight in infants born to women supplemented with protein and energy compared to infants born to women not supplemented or supplemented only with vitamin and minerals or receiving lesser amounts of supplements. Supplementation during pregnancy did not result in higher body weight in one study including women with low socioeconomic status in Philadelphia, PA, but the women were found not to be, as a group, nutritionally deprived because their dietary intake was only slightly below the recommended intake and protein intake accounted to 14.8% of the energy intake (25). Two studies (19, 28) found no difference in the birth weight of infants born to supplemented women and those born to unsupplemented women when socioeconomic status indicators were used as screening criteria. Methodological issues including better home diet containing higher protein and energy content during the experimental period compared to the baseline period (19) and heterogenous study sample may have diluted any significant difference (28). Other methodological issues include year-to-year fluctuations in home food supplies and lack of control of study participants’ supplementation intake (19).

Change in triceps skinfold during second trimester of pregnancy also appears to be an effective screening criterion. Differences between the infants’ birth weight born to supplemented and unsupplemented women were observed only among pregnant women having less than 20 µm/week increase in the triceps skinfold during the second trimester prior to supplementation (29). When the same authors included all study participants in the analysis, there was no difference in the birth weight of the infants born to supplemented and unsupplemented women (30). When skinfolds were used as a marker of maternal nutrition, mothers with lower skinfolds and receiving higher supplementation had infants 380 g heavier than mother with lower skinfolds and lower supplementation. In contrast, mothers with higher skinfolds and higher supplementation had infants that weighted only 80 g more than those born to mothers with higher skinfolds and lower supplementation (26).

There is not enough evidence to draw any conclusion but BMI may not be effective in identifying mothers who would benefit from supplementation during pregnancy. Supplementation during pregnancy leads to infants with higher birth weights (by at least 94 g) even in women with a BMI of 21 kg/m^2^ (16). Stunting was less frequent in infants born to mothers with a BMI ≥ 19.7 supplemented immediately after identification in comparison with late food supplementation, while this was not significant among infants born to mothers in the lowest half of the BMI distribution (20).

Only two studies (14, 22) have used protein intake < 40 g/day as a screening criterion, and there is not enough evidence of the validity of this criterion.

While screening criteria are important to select women who are deficit in protein and/or energy and would benefit from supplementation during pregnancy, it is also important to exclude women: 1) who would not benefit from supplementation because their recommended protein and energy intake is met by their usual food intake; 2) who would replace their usual diet with supplements; and 3) who would over-consume. Some studies in which nutrient intake was monitored with urinary nitrogen (15) or food intake methods including 24-h dietary recall (23, 28), 24-h dietary weighted records (43), 7-day dietary records (13) and home dietary survey during meal times (22) showed that the actual nutrient increment consumed by the women during pregnancy was smaller than that provided to them, ranged from 18% (23) to 70% (22) of the total energy provided in the supplement and from 52% (23) to 70% (22) of the energy provided as protein. The supplement provided during pregnancy was used to substitute the usual diet (13, 28) and thus intake from the usual diet was decreased by up to 20% (28).

### Type of supplementation

Balanced protein energy supplementation (up to 20% of energy as protein) provided during pregnancy appears to improve fetal growth and increases infants’ birth weight. Most of the studies presented in Table 1 examining balanced protein energy supplementation showed improved birth weight or length of all infants (16, 18) or in certain circumstances, i.e. when only male infants were considered in the analysis (14, 22, 23), among multiparous women (17), among women with BMI < 18.5 (17), when women were in negative energy balance (43), and among women having an increase in the triceps skinfold during second trimester < 20 µm/week (29). Amount and energy content of balanced protein energy supplementation consumed during pregnancy have also been shown to impact on fetal growth. Higher birth weight was found in infants born to women having higher intake of supplements (31) and to women consuming higher energy intake [8,780 kJ (2,100 kcal) and 11.4% of protein] compared to lower intake of supplements and lower energy intake [5,850 kJ (1,400 kcal) and 11.4% of protein] (18). Content of protein in the supplement has also been shown to influence fetal growth. Balanced protein energy supplementation during pregnancy containing 12.3% of protein produced higher fetal growth compared to supplementation containing 22.4% of protein (21). However, balanced protein energy supplementation has not been shown to improve fetal growth in some studies (13, 14, 19, 24, 30). Supplementation provided to women in positive energy balance (9), use of none or inadequate criterion to screen women who would benefit from supplementation may explain the lack of impact of balanced protein energy supplementation in fetal growth in populations with a lower prevalence of women truly at risk.

Conversely, supplements with too much protein appear to have deleterious effects on fetal growth in certain contexts. Eight studies provided high protein supplements (supplements containing more than 20% of energy as protein) to women during pregnancy (Table 1). Lower birth weight (25), increased numbers of very early premature births (28), and significant growth restriction up to 37 weeks of gestation (28) were found in infants born to women receiving supplements containing more than 20% of protein as energy compared to infants born to women in the control group. A review of 15 studies by Rush (10) found lower birth weight among infants born to women receiving supplements containing more than 20% of energy as protein compared to those born to women receiving control diet. Protein toxicity (48) and reduction of carbohydrate intake and availability (9) have been suggested to explain these findings. Some potential mechanisms for fetal amino acid toxicity which are not mutually exclusive that have been explored in animal models and are most likely to explain the observations in human studies include: 1) competitive inhibition of transport among essential amino acids across the placenta; 2) mismatch of increased fetal amino acid supply with persistently low fetal anabolic hormone concentrations; and 3) preferential utilization of increased fetal amino acids for oxidative metabolism rather than protein synthesis and accretion (49).

Positive effects on the birth weight of infants born to women receiving supplements containing more than 20% of energy content as protein have been shown in four studies (18, 21, 26, 27). The high protein content of the supplement added to the low protein content of the usual diet resulted in ideal protein content. In one of the studies (27), the supplement providing 2,900 kJ (700 kcal) and 25% of protein was added to the usual diet providing 9,380 kJ (2,244 kcal) and 13.7% of protein resulting in a net intake of 17.3% of the energy as protein. In the second study (21), the home diet provided only 8.5% of protein and the supplement just over 20% of protein. In the third study (26), the supplement containing 28% of protein was provided to women from villages with high levels of malnutrition and likely having low protein intakes. In the fourth study (18), diet with added supplement resulted in an intake of 10,240 kJ (2,450 kcal), which provided 16.5% of energy as protein. Babies with the highest birth weights (3,600 g) were born to women having 48%, 35%, and 17% of the energy intake from carbohydrate, fat, and protein, respectively (50).

No significant difference was observed in the birth weight of infants born to supplemented and unsupplemented women in four studies providing high protein (19, 38, 40), but there were some methodological issues, including masking effect of the better home diet during the time the women were receiving supplementation compared to the baseline time (19), deficit in the habitual energy intake not severe enough to impair fetal growth (38), insufficient supplement intake (40), and heterogenous study sample, as only one of seven screening criteria had to be fulfilled (13).

The source of protein in the supplement provided during pregnancy may also be important. Diet bulk including vegetable protein when used in the supplements provided to the pregnant women produced satiety before all supplement had been eaten (27). Zulu women who were supplemented with animal protein had infants with a higher birth weight (by 6.5–9.5%) than those in the placebo group or those receiving vegetable protein containing the same content of iron (27). However, lower uptake of iron from vegetable protein in the supplements provided to the mothers may have contributed to the lower birth weight compared to the birth weight of infants born to mothers receiving animal protein, which is highly absorbable.

As with too much protein, diets containing a high percentage of energy from carbohydrate had a greater negative effect on fetal growth. Women with low triceps gain supplemented with carbohydrate only [1,780 kJ (425 kcal) from syrup glucose] gave birth to infants with lower birth weight (2,900 g) compared to those infants (3,020 g) born to women not receiving any supplement during pregnancy (29).

The findings from these studies suggest that fetal growth is influenced more by the total diet intake, including the usual diet and the supplement, than the supplement content consumed by the women.

Most pregnant women will probably need a total of 9,200–12,120 kJ (2,200–2,900 kcal) per day. The extra energy needed is 1,420 kJ (340 kcal) and 1,890 kJ (452 kcal) in the second and third trimester, respectively (51).

It is important to have a good understanding of the usual diet of the women in order to identify if and what supplementation is needed during pregnancy. It is also important to know the dietary practices (52). Another important aspect to be considered is when to start supplementation during pregnancy, which is discussed in the next section.

### Time to start the supplementation

There is no consensus on the best time to start supplementation during pregnancy to optimize fetal growth. Nutritional status of the woman during the preconception period may be a greater determinant of fetal growth than nutritional status during the latter part of pregnancy (34). Some studies showed that supplementation should start before a woman becomes pregnant, perhaps during the postpartum period from the previous child (34). The higher the amount of supplements consumed by women during first pregnancy, lactating period and second pregnancy, the higher the birth weight of their second infants (31). If the woman is well nourished at conception and during early pregnancy, maternal physiological and metabolic adjustments to pregnancy proceed in a normal fashion (34). Conversely, higher proportions of LBW infants have been observed among short (less than 150 cm) (53, 54), malnourished (BMI < 18.5) (24) and anemic women (6). When the women are both anemic and malnourished, their babies’ birth weight are even smaller (6). There is enough evidence to suggest that anemic and malnourished women are likely to benefit from balanced protein energy supplementation. However, even in conditions of undernutrition and consuming only 60% of the recommended dietary allowance, pregnant women were able to maintain a positive energy balance (43). A modest 4 kg weight gain during pregnancy still resulted in infants with adequate birth weight (BW > 2,500 g) (9). Institute of Medicine recommended weight-gain ranges are 11.5–16 kg for normal weight women and 12–18 kg for underweight women. Mobilization of fat (9) and energy-sparing metabolic adjustments during pregnancy (16) have been suggested as the explanation for infants with normal birth weight being born to women with inadequate weight gain during pregnancy. Composition of maternal weight gain during pregnancy also seems to contribute to fetal growth (21, 48). Women who put on less fat (triceps skinfold increased < 20 µm/week between 18 and 28 weeks of pregnancy) had infants with higher birth weight (3,350 g versus 2,940 g) compared to women who put on more fat (triceps skinfold increased > 20 µm/week between 18 and 28 weeks of pregnancy) (29). Fat mobilization may be the underlying mechanism, as described above.

Some studies showed that supplementation should start as early as possible (20, 28, 31, 44, 46, 47). Longer pregnancy (1.4 week longer) (26) and small benefits on infants’ performance were observed when women were supplemented early in pregnancy (47). However, studies from Africa show that fetal growth is severely retarded during late gestation (9) and therefore this may be the period most amendable to intervention (16). Compared to a well-nourished population, Gambian babies were 250 and 600 g smaller at week 35 and at term, respectively (16).

It is not known if the frequency or the time of the day to provide supplementation during pregnancy influences fetal growth but it is believed that providing a supplement during a long period without food would introduce a glucose peak and consequently increase insulin levels (9). Insulin promotes mitosis, increases glucose uptake and oxidation in fetal tissues and alters concentrations of IGF-1 in utero; all of which affect fetal growth (6).

In summary, there is no consensus on the best time to start supplementation during pregnancy to optimize fetal growth. Nutritional status of the woman during the preconception period may be a greater determinant of fetal growth than nutritional status during the latter part of pregnancy. Conversely, fetal growth is severely retarded during late gestation and therefore this may be the period most amendable to intervention.

## Conclusions

Overall, LBW is a major problem throughout the developing world. Important maternal determinants of the fetal growth are maternal nutritional status and nutrition during pregnancy especially if the woman is malnourished and enters pregnancy without adequate reserves. Positive outcomes in infants and women cannot be expected if the supplementation is not needed. There is no consensus on the most effective means of screening women for dietary intervention programs. Change in triceps skinfold during second trimester of pregnancy appears to be an effective screening criterion. BMI < 18.5 kg/m^2^ does not seem as effective as skinfolds to identify mothers needing supplementation during pregnancy. Socioeconomic status or living conditions may also provide a good indication of women who would benefit from supplementation during pregnancy. Balanced protein energy supplementation (up to 20% of energy as protein) provided during pregnancy appears to improve fetal growth and increases infants’ birth weight. Conversely, supplements with too much protein or too much carbohydrate appear to have deleterious effects on fetal growth in certain contexts. Total diet intake, including the usual diet and the supplement, rather than the supplement content consumed by the women during pregnancy is crucial to fetal growth. There is also no consensus on the best time to start supplementation. While most of the fetal weight gain occurs in the third trimester, nutritional status of the woman during the preconception period may be a more important determinant of fetal growth than nutritional status during the latter part of pregnancy. Strong quality studies examining criteria to screen women who would benefit from supplementation, time to start supplementation and type of supplements are warranted.
